# Annual Election of the Philadelphia Medical Society

**Published:** 1845-01

**Authors:** Job Haines

**Affiliations:** Junior Recording Secretary


### PHILADELPHIA MEDICAL SOCIETY.

At the annual election of this Society, held at their Hall on the 4th inst., the following members were chosen officers for the ensuing year, viz:

*President.*—R. M. Huston, M. D.

*Vice Presidents.*—Benjamin H. Coates, M. D., Henry Bond, M. D.

*Treasurer.*—John Wiltbank, M. D.

*Corresponding Secretaries.*—Joseph Warrington, M. D., Isaac Parrish, M. D.

*Senior Recording Secretary.*—John J. Reese, M. D.

*Orator.*—Henry S. Patteson, M. D.

*Librarian.*—Nathan D. Benedict, M. D.

*Curators.*—Aaron D. Chaloner, M. D., Edmund Lang, M. D.

Job Haines, *Junior Recording Secretary.*

